# A novel multiplex-protein array for serum diagnostics of colon cancer: a case–control study

**DOI:** 10.1186/1471-2407-12-393

**Published:** 2012-09-07

**Authors:** Stefanie Bünger, Ulrike Haug, Maria Kelly, Nicole Posorski, Katja Klempt-Giessing, Andrew Cartwright, Stephen P Fitzgerald, Vicki Toner, Damien McAleer, Timo Gemoll, Tilman Laubert, Jürgen Büning, Klaus Fellermann, Hans-Peter Bruch, Uwe J Roblick, Hermann Brenner, Ferdinand von Eggeling, Jens K Habermann

**Affiliations:** 1 Laboratory for Surgical Research, Department of Surgery, University of Lübeck, Ratzeburger Allee 160, D-23538, Lübeck, Germany; 2 Division of Preventive Oncology, National Center for Tumor Diseases/German Cancer Research Center (DKFZ), Im Neuenheimer Feld 280, 69120, Heidelberg, Germany; 3 Division of Clinical Epidemiology and Aging Research, German Cancer Research Center (DKFZ), Bergheimer Str. 20, 69115, Heidelberg, Germany; 4 Randox Laboratories GmbH, Wilhelmstr, 147a, 42489, Wulfrath, Germany; 5 Core Unit Chip Application, Institute of Human Genetics, Jena University Hospital, Leutragraben 3, 07743, Jena, Germany; 6 Department of Internal Medicine I, University Hospital of Schleswig-Holstein, Campus Lübeck, Ratzeburger Allee 160, 23528, Lübeck, Germany

**Keywords:** Multiplex protein array biochip, Colon cancer screening, Serum diagnostics, High-throughput seromics, IL-8, CEA, CRP

## Abstract

**Background:**

More than 1.2 million new cases of colorectal cancer are reported each year worldwide. Despite actual screening programs, about 50% of the patients are diagnosed at advanced tumor stages presenting poor prognosis. Innovative screening tools could aid the detection at early stages and allow curative treatment interventions.

**Methods:**

A nine target multiplex serum protein biochip was generated and evaluated using a training- and validation-set of 317 highly standardized, liquid nitrogen preserved serum samples comprising controls, adenomas, and colon cancers.

**Results:**

Serum levels of CEA, IL-8, VEGF, S100A11, MCSF, C3adesArg, CD26, and CRP showed significant differences between cases and controls. The largest areas under the receiver operating characteristics curve were observed for CEA, IL-8, and CRP. At threshold levels yielding 90% specificity, sensitivities for CEA, IL-8 and CRP were 26%, 22%, and 17%, respectively. The most promising marker combinations were CEA + IL-8 reaching 37% sensitivity at 83% specificity and CEA + CRP with 35% sensitivity at 81% specificity. In an independent validation set CEA + IL-8 reached 47% sensitivity at 86% specificity while CEA + CRP obtained 39% sensitivity at 86% specificity. Early carcinomas were detected with 33% sensitivity for CEA + IL-8 and 28% for CEA + CRP.

**Conclusions:**

Apart from CEA, IL-8, and CRP, the screening value of additional blood markers and the potential advantage of combining serum biochip testing with fecal occult blood testing needs to be studied. Multiplex biochip array technology utilizing serum samples offers an innovative approach to colorectal cancer screening.

## Background

Colon cancer ranks among the most frequent malignancies and is the fourth leading cause of cancer-related death worldwide [1,2]. Detection of colon cancer at early stages is critical for curative treatment interventions: although the 5-year disease-free survival for International Union Against Cancer (UICC) stage I tumors exceeds 90%, this rate is reduced to 63% in UICC III and < 5% in UICC IV carcinomas [3]. Yet, despite the implementation of current screening programs about 50% of these malignancies are detected at advanced tumor stages. Therefore, tools and methodologies that allow early colon cancer detection directly impact on survival times. In present clinical practice, screening for cancer and premalignant polyps of the colon is based on clinical examination, the detection of fecal occult blood, and on sigmoidoscopy or colonoscopy [4,5]. The successful implementation of these screening procedures has contributed to a reduction of disease-associated mortality of colon carcinomas [6]. The persistent delay in diagnosis and the associated high mortality rate are attributable to a low compliance to some screening tests and to the low sensitivity of other tests [7]. An optimal, alternative screening test would be relatively non-invasive and achieve high patient compliance, fulfil excellent analytical performance regarding sensitivity and specificity and still be robust and cost-effective. Such a test can be envisioned if changes in the composition of serum proteins could indicate specific diseases and/or disease stages. Comprehensive serum proteome profiling for tumor-specific markers has therefore become a field of intensive research. For colon cancer screening the application of serological testing has not been established so far, even though very promising candidate markers have been reported. Based on a thorough literature review, the most promising markers were identified. By using preoperative serum levels for CRC diagnosis with sensitivity and specificity of 90% was reported for protein 26 (CD26) [8]. Other diagnostic approaches based on the detection of carcinoembryonic antigen (CEA), vascular endothelial growth factor (VEGF) [9], macrophage colony-stimulating factor (M**-**CSF) [10], Nicotinamide N-methyltransferase (NNMT) [11], or C-reactive protein (CRP) concentrations [12] reached either a high sensitivity or specificity. Furthermore, within our consortium, Interleukin 8 (IL-8) [13], Calgizzarin (S100A11) [14] and complement component 3a (C3adesArg) [15] serum levels were determined as potentially promising biomarkers for CRC [14]. However, most of the markers are yet to be validated in well defined, large screening studies. Hereby, the measurement of a disease specific panel of markers could outperform the measurement of individual markers regarding sensitivity and specificity. In addition, this approach could provide a more comprehensive reflection of molecular networks and pathophysiological conditions of diseases.

Biochip array technology allows multiplex determination of multiple biomarkers from a single sample [16,17]. This is also relevant when volumes of clinical samples are limited. Implementations of this technology in clinical settings have been reported for different biochip arrays including cytokines [13], cerebral and cardiac arrays [18,19], adhesion molecules [20], and also detection of drug abuse [21].

The aim of this study was to apply biochip array technology to colon cancer screening. For this purpose, a biochip array was designed and developed for the multiplex determination of nine serum markers allowing for low inter-analysis variability, decreased workload and faster processing time as well as lower costs due to high-throughput automation. The performance of the two biochip arrays for colon cancer screening was then evaluated in a training and a validation set consisting of 317 highly standardized, liquid nitrogen preserved serum samples.

## Methods

### Study group

This study comprised 317 serum samples that were randomly selected from 3,700 serum samples collected at the University Clinic Schleswig-Holstein, Campus Lübeck, Germany, between 2007 and 2011. This serum collection belongs to the biomaterial bank *ColonBiomics* being an integral part of the Surgical Center for Translational Oncology – Lübeck (SCTO-L), University of Lübeck, and the DKH e.V. funded network *North German Tumorbank of Colorectal Cancer* (ColoNet, #108446). Serum samples were collected adhering to the guidelines of the local ethical review board (Medical University of Lübeck, #07-124) and according to strict standard operation procedures. Serum samples of healthy control patients as well as cancer patients were both taken after bowl-preparation and prior to colonoscopy or oncologic resection. The 317 samples comprised 164 patients with histological confirmed colon cancer (96 men and 68 women), 34 patients with colon adenomas (18 men and 16 women), and 119 healthy controls (52 men and 67 women) (Table 1). Out of this cohort we defined a training set of 52 healthy controls and 81 patients with colon malignancy and an independently collected, non-overlapping validation set of 50 controls and 83 colon carcinoma samples. For control patients, blood samples were obtained before full colonoscopy, which confirmed that no signs of inflammatory, benign, premalignant or malignant lesions were present in this cohort. For cancer patients, blood samples were obtained before neoadjuvant chemo- or radiotherapy and/or surgery. Detailed clinical data of the patient cohort are summarized in Tables 1, 2, 3.

**Table 1:** Clinical data of the study group

**Summary of clinical data of the study group consisting of colon cancer patients, pre-malignant adenoma patients and healthy control patients**					
**Parameter**	**Value**	**Colon cancer (CC) patients**	**Healthy control (H) patients**	**Adenoma (A) patients**	**P-value**
		**(n = 164)**	(n = 119)*	**(n = 34)**	
**Sex**	Female	68 (41.5%)	67 (56.3%)	16 (47.1%)	0.738
	Male	96 (58.5%)	52 (43.7%)	18 (52.9%)	
**Age**	Range	40.5 – 99.1	19.5 – 90.8	28.1 – 86.1	0.144 (A vs H)
**(years)**	Mean	69.59	62.41	63.65	0.004 (CC vs H)

*All healthy patients received a full colonoscopy.

**Table 2:** Clinical data of the study group

**Summary of clinical data of colon cancer patients**			
**Parameter**	**Value**	**Colon cancer patients**	**%**
**UICC stage**	1	30	18.3
	2	50	30.5
	3	59	36.0
	4	25	15.2
**T status**	1	10	6.3
(tumor size)	2	26	16.5
	3	102	64.6
	4	20	12.7
**N status**	0	45	52.3
(nodal status)	1	22	25.6
	2	19	22.1
**M status**	0	40	45.5
(distant metastasis)	1	34	38.6
	2	14	15.9
**Tumor**	G1	61	74.4
**grading**	G2	13	15.9
	G3	18	9.7

**Table 3:** Clinical data of the study group

**Summary of clinical data of colon adenoma patients**						
**Patient #**	**Sex (female = f male = m)**	**Age (years)**	**Dysplasia**	**Histology**	**Adenoma size (cm)**	**Advanced (A) or non-advanced (Non-A)**
1	f	71.0	low	Tubular	0.3	Non-A
2	f	28.1	low	Tubular	0.7 - 1.0	A
3	m	53.0	low	Tubular	1.0	A
4	m	72.0	low	Tubular	1.2	A
5	m	71.8	low	Tubular	1.0 - 1.5	A
6	m	58.0	low	Tubular	0.3 - 0.4	Non-A
7	m	67.1	N/A	Tubular	0.6	Non-A
8	f	45.9	low	Tubular	0.7	Non-A
9	f	81.5	low	Tubular	0.1 - 0.3	Non-A
10	m	54.0	high	Tubulovillous	0.85	A
11	m	73.8	high	Tubulovillous	N/A	A
12	f	79.8	high	Tubulovillous	0.8	A
13	f	49.4	high	Tubulovillous	4.5	A
14	m	54.7	high	Tubulovillous	2.5	A
15	m	79.6	low	Tubulovillous	N/A	A
16	m	63.3	low	Tubulovillous	2.0	A
17	f	75.1	low	Tubulovillous	N/A	A
18	f	52.6	low	Tubulovillous	N/A	A
19	f	64.1	low	Tubulovillous	1.0	A
20	f	73.5	low	Tubular	N/A	Non-A
21	m	83.1	low	Tubular	2.5	A
22	m	56.4	low	Tubular	N/A	Non-A
23	m	65.8	low	Tubular	0.1 - 0.2	Non-A
24	m	69.5	low	Tubular	N/A	Non-A
25	f	32.3	low	Tubular	N/A	Non-A
26	f	62.4	low	Tubular	0.1 - 0.2	Non-A
27	f	64.5	low	Tubular	N/A	Non-A
28	m	68.3	low	Tubulovillous	2.4	A
29	f	45.1	low	Tubulovillous	6.0	A
30	f	67.8	low	Tubulovillous	3.0	A
31	f	86.1	low	Tubulovillous	1.0	A
32	m	63.2	low	Tubulovillous	N/A	A
33	m	63.1	low	Tubulovillous	N/A	A
34	m	68.1	low	Tubulovillous	N/A	A

An additional cohort of 400 serum samples was used for a pilot study in order to optimize the prototypes of the biochips. This cohort is part of a colon cancer screening cohort established at the German Cancer Research Center (DKFZ) and is described in detail in Additional file 1: Table S1.

### Sampling

All venous blood samples were obtained using serum gel-monovettes (#01.1602, Sarstedt AG & Co, Nümbrecht, Germany) and centrifuged at 1,550× g for 10 min at 4°C to separate the serum. Aliquots of serum samples were stored at −196°C within 30 min after venous puncture. Samples were thawed on ice before multiplex assessment on the newly designed biochips.

### Development of the CRCS Multiplex Biochips

Both chips were manufactured according to standards described [16,17]. Target analytes for the CRCS I and II arrays were based on an extensive review of the literature [22] and own experimental data [14,15,13]. Design input requirements were determined following literature specifications. The product was manufactured and validated on 100 serum samples according to Randox Laboratories’ manufacturing guidelines and procedures. The validation followed a series of approved standard operating procedures. The sensitivity, accuracy and precision of each assay were determined. Sensitivity was evaluated to determine the lowest concentration that could be accurately detected for an assay. Precision was assessed both within runs (intra) and between runs (inter). Three samples of known concentrations which span the assay range were assessed 20 times as a measure of the intra-assay precision. The precision is expressed as the co-efficient of variation (%) over the 20 replicates. Inter-assay precision implies the assessment of these 3 samples in duplicate over 10 separate runs. Again the precision was assessed as the co-efficient of variation (%) over 20 replicates. Deviation of ≤ 15% is acceptable for both intra- and inter-assay precision. Current performance data for both CRCS I and CRCS II are shown in Additional file 2: Table S2.

### Optimization of the CRCS Multiplex Biochips

Once the biochip prototypes were manufactured, a pilot study of 400 serum samples (100 controls, 100 early adenomas, 100 advanced adenomas, 100 colorectal carcinomas, Additional file 1: Table S1) was conducted with the developed biochip arrays. Based on the results of the pilot study, incubation times were changed from 2 x 30 min to 2 x 60 min in order to improve sensitivity. In addition, changes to some assay ranges were implemented for the subsequent manufacturing process: C3adesArg was changed from 0 – 1.8 μg/mL to 0 – 600 ng/mL, M-CSF from 0 – 1 ng/mL to 0 – 500 pg/mL, NNMT from 0 – 50 ng/mL to 0 – 70 ng/mL and S100A11 changed from 0 – 50 ng/mL to 0 – 200 ng/mL.

### Determination of biomarker serum levels

Two biochip platforms were designed for the simultaneous quantitative detection of the nine-biomarkers biochip array in combination with the *Evidence Investigator* analyser (Randox Laboratories Ltd., Crumlin, UK). Due to different assay- and detection ranges of some biomarkers, the nine chosen serum markers had to be subdivided onto two biochip platforms: One biochip (CRCSI) comprised C3desArg, CD26 and CRP, and the other biochip (CRCSII) incorporated IL-8, CEA, VEGF, M-CSF, S100A11 and NNMT. Both biochips were manufactured according to standards described [16], and assay ranges and sensitivities for each analyte were measured (Additional file 2: Table S2).

Both biochip arrays are based on simultaneous chemiluminescent sandwich immunoassays. Capture antibodies, specific for each biomarker are bound to the biochip surface defining arrays of test sites. The biochip functions as the solid phase and reaction vessel for the immunoreactions. The assays were applied to the *Evidence Investigator* analyser. The system allows handling of up to 54 samples (6 x 9 wells) in biochip carriers (Additional file 3: Figure S1). Three controls and nine point calibration curve were run in parallel with 42 patient samples. The inclusion of controls and calibration samples avoids the need for technical replicates for patient samples. A total of 100 μL of 1:200 diluted serum for CRCSI and 90 μL undiluted serum samples for CRCSII were applied. Upon completion of the immunoreactions, the chemiluminescent signal in each test site was detected with a super cooled charged coupled device (CCD) incorporated with the system data automatically processed. The detailed protocol for biochip incubation and processing can be found as Additional file 4.

The analyser routinely assesses the quality of assay performance and generates calibration curves as described by FitzGerald *et al.*[17]. The analyte’s concentration present in the sample was calculated automatically using generated calibration curves (*Evidence Investigator Software version 1.4*).

### Statistical analysis

Using SPSS Statistics version 19 (*IBM Corporation, Somer, NY*) and SAS version 9.1 (*SAS Institute Inc., Cary, North Carolina, USA*), the serum levels of the individual markers in colon cancer cases, colon adenoma cases and healthy controls were described with respect to median levels, interquartile ranges and overall ranges. Nonparametric tests to compare median serum levels between different patient groups were applied. The correlation between age and sex and the serum levels of the markers as well as correlations between the single markers was assessed using Spearman’s correlation coefficient. Furthermore, receiver-operating characteristics (ROC) curves were constructed and the area under the ROC curves (AUC) with 95% confidence intervals (CI) for each single marker was calculated in relation to the discrimination between colon cancer cases and controls. We also calculated the AUC for the combination of all markers using logistic regression analysis and compared this AUC to the AUC of the single markers.

While logistic regression modelling focuses on the overall AUC, we focused - in a next step – on the part of the AUC that is most relevant for the screening setting. Considering sensitivities at levels of specificity that are typically required in the screening setting (≥ 90%), we selected markers showing the best discriminative power. The training set was then used to determine a threshold level (yielding a specificity of 90%) for each of these selected markers. The sensitivity of the marker was then calculated at this threshold level followed by an assessment of which marker combination could have a beneficial affect regarding test performance. Concerning the combination, individuals with at least one marker above the individual marker threshold were classified as positive. Marker combinations were considered as beneficial if they increased sensitivity compared to the single markers without substantially lowering the specificity. This approach reaches for a high specificity in order to keep the number of false-positive tests limited.

Finally, this algorithm regarding threshold levels and promising marker combinations that were developed in the training set was applied to an independent validation set including early stage carcinomas and adenomas.

## Results

Here we report a comprehensive evaluation of serum proteins in an effort to validate screening biomarkers for colon tumors. Two multiplex biochips (CRCSI and CRCSII) were manufactured consisting of altogether nine serum markers and evaluated their performance for detecting colon cancer in a minimally-invasive, patient-friendly and reliable fashion. Biochips and targets were simultaneously evaluated in a highly standardized serum collection of cancer and control samples stored at −196°C within 30 min after phlebotomy following strict standard operation procedures.

### Evaluation of single markers

**Figure 1:**
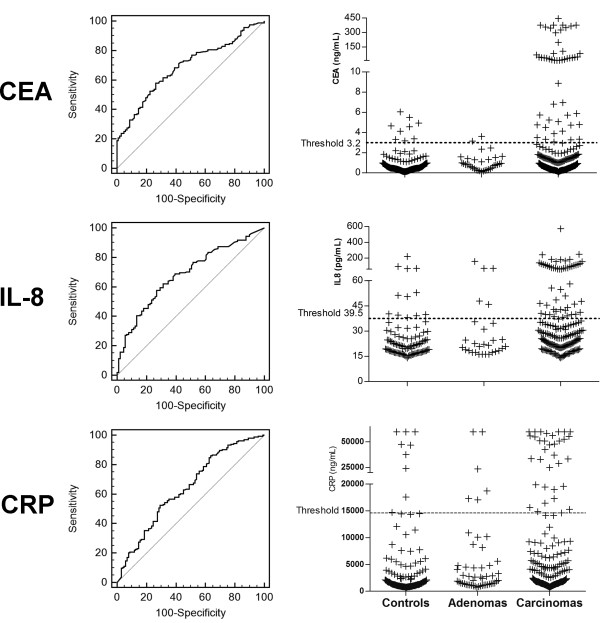
**Diagnostic performance of single markers for early detection of CRC.** Receiver operating characteristics curves regarding the discrimination between colon cancer and controls for CEA, IL-8 and CRP as single biomarkers with single sample dot plots including specification of thresholds.

The distribution of serum levels of all nine markers is described in Table 4. Serum levels of CEA, IL-8, VEGF, S100A11, C3adesArg, CD26, MCSF and CRP showed significant differences between colon cancer cases and controls (P < 0.05). The ROCs of the single markers are shown in Figure 1 and the AUCs are listed in Table 5. The three largest AUCs were observed for CEA (0.69), IL-8 (0.68), and CRP (0.64). CEA, IL-8 and CRP were also the markers whose sensitivities were significantly different from the false-positive rate at levels of specificity that are typically required in a screening setting (≥ 90%).

**Table 4:** Serum level of nine serum biomarkers in colon cancer patients in comparison to adenoma cases and healthy control patients

		**Colon cancer patients *(n = 164*) **	**Control patients *(n = 119)***	**Adenoma patients *(n = 34)***
**CEA ‡ (ng/mL)**	median	1.26	0.51	0.82
	inter-quartile range	0.54 – 4.01	0.36 -1.09	0.41 – 1.34
	range	0.08 - 444.32	0.14 - 6.05	0.12 - 3.62
	p-value	< 0.0001		
**IL-8 ‡ (pg/mL)**	median	27.46	20.47	19.97
	inter-quartile range	20.20 – 42.36	16.78 – 26.21	16.20 – 26.52
	range	0.00 - 572.58	0.00 - 221.15	0.00 - 158.39
	p-value	< 0.0001		
**VEGF ‡ (pg/mL)**	median	77.99	59.94	67.86
	inter-quartile range	46.78 – 120.94	34.99 – 98.70	42.95 – 91.41
	range	5.02 - 350.38	7.54 - 427.56	10.68 - 178.72
	p-value	0.02		
**M-CSF ‡ (pg/mL)**	median	8.27	7.18	6.65
	inter-quartile range	6.70 – 12.76	5.31 – 10.26	5.47 – 8.58
	range	2.26 - 58.23	2.99 - 72.86	3.50 - 30.55
	p-value	0.02		
**S100A11 ‡ (ng/mL)**	median	12.14	10.89	11.93
	inter-quartile range	9. 39 – 15.46	7.97 – 14.18	9.61 – 16.05
	range	4.37 - 177.68	3.99 - 105.25	6.69 - 56.93
	p-value	0.04		
**NNMT (ng/mL)**	median	1.48	1.46	1.63
	inter-quartile range	0.72 – 4.23	0.67 – 4.60	0.64 – 7.82
	range	0.25 - 70.00*	0.27 - 70.00*	0.28 - 70.00*
	p-value	> 0.05		
**C3a desArg ‡ (ng/mL)**	median	708	808	660
	inter-quartile range	514 – 906	538 – 1134	344 – 1216
	range	140 - 6114	150 - 3316	200 - 3282
	p-value	0.04		
**CD26 ‡ (ng/mL)**	median	442	550	614
	inter-quartile range	378 – 582	428 – 698	494 – 750
	range	184 - 1352	0.00 - 1034	82 – 3980*
	p-value	0.0002		
**CRP ‡ (ng/mL)**	median	4018	2086	2020
	inter-quartile range	1718 – 9238	1188 – 5260	1208 – 4768
	range	778 - 59800*	726 - 59800*	902 - 59800*
	p-value	0.006		

*Detection Limit, ‡ Comparison of medium serum levels between colon cancer patients and control patients p < 0.05.

**Table 5:** Diagnostic performance of nine serum biomarkers area under the receiver operating characteristics curve for all nine analyzed biomarkers in single analysis for colon cancer cases versus controls

***Biomarker***	AUC* (95% confidence interval)
***CEA***	***0.687***
	***(0.627 – 0.742)***
***IL-8***	***0.684***
	***(0.622 – 0.741)***
***CRP***	***0.640***
	***(0.579 – 0.698)***
***CD26***	***0.639***
	***(0.578 – 0.696)***
*S100A11*	*0.597*
	*(0.536– 0.657)*
*VEGF*	*0.596*
	*(0.534 – 0.656)*
*C3adesArg*	*0.591*
	*(0.529 – 0.651)*
*M-CSF*	*0.583*
	*(0.522 – 0.643)*
*NNMT*	*0.521*
	*(0.459 – 0.582)*

* Area under the receiver operating characteristics curve.

### Evaluation of marker combinations

In a first step, we assessed whether the combination of all markers indicates an advantage in terms of increasing the overall AUC compared to AUC of the single markers. The AUC of all markers combined as determined by logistic regression was 0.75, which was not statistically significantly different from the largest AUC among the single markers (i.e., the AUC of CEA). While logistic regression modelling focuses on the overall AUC, we focused - in a next step – on the part of the AUC that is most relevant for the screening setting. We assessed whether there was a combination of markers that yields an increased sensitivity when focusing on levels of specificity that are typically required in a screening setting (≥ 90%). For that purpose, we selected CEA, IL-8 and CRP whose sensitivities were significantly different from the false-positive rate at these levels of specificities and developed a test algorithm for their combination as described below.

### Development of test algorithm in the training set

Sera from 52 healthy controls and 81 patients with colon cancer were used to develop a test algorithm. We determined threshold levels yielding a specificity of 90% for CEA, IL-8, and CRP. For CEA the threshold was at 3.2 ng/mL, for IL-8 at 39.5 pg/mL, and for CRP at 14,600 ng/mL. Based on these threshold levels (yielding a specificity of 90%), CEA showed a sensitivity of 26% (95%CI: 17-37%), IL-8 of 22% (95%CI: 14-33%), and CRP of 17% (95%CI: 10-27%; Figure 1).

We then assessed which combination of these markers could increase the assay sensitivity without substantially lowering its specificity. The following two combinations showed the best results: CEA + IL-8 reached a sensitivity of 37% (95%CI: 27-48%) at a specificity of 83% (95%CI: 70-92%) and CEA + CRP obtained a sensitivity of 35% (95%CI: 24-46%) at a specificity of 81% (95%CI: 67-90%). The combination of IL-8 + CRP yielded both a lower sensitivity and a lower specificity compared to CEA + IL-8 and the combination of all three markers decreased the specificity to 73%, but increased the sensitivity by only 4% compared to CEA + IL-8.

### Evaluation of the test algorithm in an independent validation set

To exclude fortuitous separation of the colon carcinoma cases from healthy controls in the training set, the test algorithms described above were then applied to the validation set of 133 serum samples. The marker combination of CEA + IL-8 reached a sensitivity of 47% (95%CI: 36-58%) at a specificity of 86% (95%CI: 73-94%), while CEA + CRP obtained a sensitivity of 39% (95%CI: 28-50%) at a specificity of 86% (95%CI: 73-94%). Thus, the diagnostic performance for marker combinations of CEA, IL-8 and CRP proved to be slightly better in the validation set. In comparison, the performance of CEA alone would have reached a sensitivity of only 37% (95%CI: 29-44%) at the same level of specificity at 86%.

### Application of both marker combinations

The marker combinations of CEA + IL-8 and CEA + CRP each falsely determined seven of 52 healthy controls to be malignant. Out of these seven samples, four were falsely identified by both marker combinations. For colon carcinomas, the combination CEA + IL-8 correctly recognized 39 and CEA + CRP 32 of 81 patients correctly as malignant. A total of 31 cases overlapped between both combinations. Combining CEA + IL-8 with CEA + CRP analysis showed a minimal increased sensitivity of 48% as compared to 47% for CEA + IL-8 alone, while specificity decreased from 86% to 80%. Thus, combining both marker combinations did not present a relevant advantage regarding test performance (Additional file 5: Table S3).

### Serum levels and test performance for early stage carcinomas

**Figure 2:**
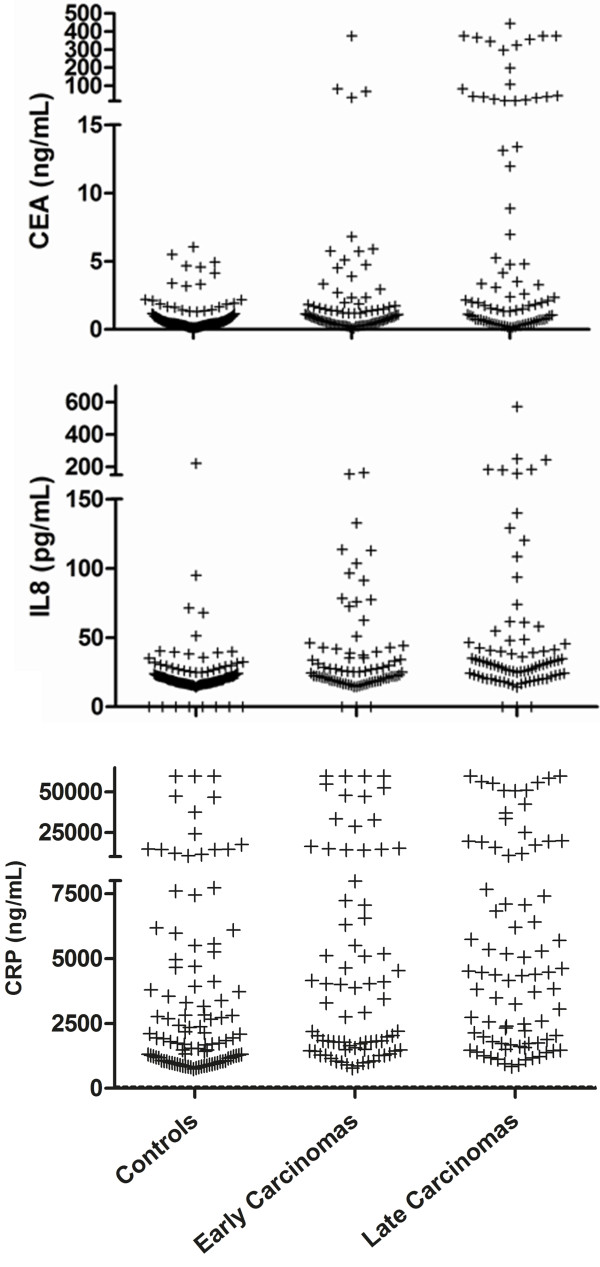
**Detection of early colorectal carcinoma.** Dot plots for serum level of CEA, IL-8 and CRP in control patients in comparison to early and late colon carcinoma.

When comparing serum levels of CEA and IL-8 between all early stage (UICC stages I and II, n = 80) and late stage colon carcinomas (UICC stages III and IV, n = 84), the median serum level for CEA was 8.00 ng/mL in early stage and 47.97 ng/mL in late stage carcinomas (P = 0.0051). The serum levels for IL-8 were 35.78 pg/mL in early stage and 52.76 pg/mL in late stage carcinomas (P = 0.0380). For CRP, median serum level was 3,366 ng/mL in early stage and 4,380 ng/mL in late stage carcinomas (P > 0.05) (Figure 2). Applying the afore described test algorithm to 80 early stage carcinomas of the combined training and validation set, the sensitivity was 33% (95%CI: 22-44%) for the combination of CEA + IL-8 and 28% (95%CI: 18-39%) for the combination of CEA + CRP, each at a specificity of 86% (95%CI: 73-94%). Looking at the performance of the marker combinations in more detail by each UICC stage separately, at UICC I (19 cases) the combination of CEA + IL-8 reached a sensitivity of 21% (95%CI: 8-55%) while CEA + CRP yielded 5% (95%CI: 0-26%). For UICC II (21 cases), the sensitivity increased to 48% (95%CI: 26-70%) for both marker combinations (CEA + IL-8 and CEA + CRP). At UICC III (28 cases), the sensitivity was 39% (95%CI: 22-59%) for CEA + IL-8 and 29% (95%CI: 13-49%) for CEA + CRP. The highest sensitivity was reached at UICC IV (15 cases) with 93% (95%CI: 68-100%) for CEA + IL-8 and 87% (95%CI: 60-98%) for CEA + CRP.

### Serum levels and test performance for adenomas

We had collected sera from 34 patients in whom the presence of an adenoma was determined by colonoscopy (Table 3). The biochip results for all adenomas are included in Figure 1. The median CEA serum levels of 34 patients with adenomas (0.82 ng/mL) were lower than the levels observed in all patients with invasive carcinomas (1.26 ng/mL). However, the levels were significantly higher than the median serum levels of the healthy controls (0.55 ng/mL, P = 0.0485). For IL-8 the median serum level for patients with adenomas (19.97 ng/mL) was also lower than the levels observed in all patients with invasive carcinoma (27.46 ng/mL) and in all healthy controls (20.74 ng/mL, both P > 0.05). Similar results showed CRP with low median levels of 2,020 ng/mL for adenoma patients and higher levels of 4,018 ng/mL for carcinoma patients and 2,086 ng/mL for healthy controls (both P > 0.05). The afore described test algorithms were applied to assess the assays potential for discriminating patients with adenomas from healthy controls. First, adenomas were analysed separately by stage of progression: The sensitivity for the combination of CEA + IL-8 was 17% (95%CI: 2-48%) for non-advanced and 23% (95%CI: 8-45%) for advanced adenomas. For the combination of CEA + CRP, non-advanced adenomas were detected with a sensitivity of 17% (95%CI: 2-48%) and advanced stage adenomas with 14% (95%CI: 3-35%) (all at a specificity of 86% (95%CI: 73-94%)). The combination of CEA + IL-8 recognized two of 12 non-advanced and five of 22 advanced adenomas and the combination of CEA + CRP detected also two of 12 non-advanced and three of 22 advanced adenoma patients correctly. For the non-advanced adenomas, one patient, which was positive detected by CEA + IL-8 was also detected by CEA + CRP, for the advanced adenomas also one positive patient overlapped between both marker combinations. Second, all adenomas were analysed as whole group: The sensitivity for the combination of CEA + IL-8 was 18% (95%CI: 7-35%) and 15% (95%CI: 5-31%) for the combination of CEA + CRP, (both at a specificity of 86% (95%CI: 73-94%)). The combination of CEA + IL-8 recognized six and the combination of CEA + CRP detected five of 34 adenoma patients correctly. A total of two adenoma patients overlapped between the two combinations. Application of both marker combinations simultaneously showed a minimally increased sensitivity of 26%, while the specificity decreased to 80%.

### Correlation among biomarker levels and clinicopathological features

Individuals in the control groups were younger (62.4 years) than those afflicted with colon cancer (69.6 years) (P = 0.004). No differences were observed between adenoma cases and controls regarding the age of the patients (P = 0.144). Sex was distributed equally between all groups (P = 0.738). In order to explore whether the serum levels of the nine tested markers showed a correlation with age or sex, Spearman´s correlation coefficients were calculated. There was no relevant correlation between the nine tested markers and age or sex. However, serum levels of M-CSF correlated with age within the control-, adenoma- and carcinoma group (Spearman’s correlation coefficient > 0.35, P < 0.05) (Additional file 6: Table S4).

## Discussion

This is the first study to report the development of a serum biochip array for the simultaneous assessment of nine serum biomarkers for clinical application to colon cancer screening in a large and highly standardized serum sample cohort.

### Performance of the developed CRCS biochip array technology

The biochip-array-technology represents a highly standardized technique for cancer research. Important and interesting at this point is the simultaneous determination of multiple analytes in a single patient sample, whereas ELISAs are limited to single analyte determinations per sample. Therefore, economics of consumables and most notably sample volume requirements are much lower for the biochip assay (100 μL total for evaluating nine analytes simultaneously, equal to 11.1 μL per analyte), which is an essential advantage concerning precious clinical samples. In addition, the throughput by a single operator is much higher for the multiplex assay than for ELISAs: in our set up 84 patient samples can be processed for nine analytes within four hours under highly standardized conditions. Furthermore, a scale up of the multiplex assay to even higher throughput is easily possible by using semi- or full-automation Evidence Multiplex Analyzers, which have the capacity to perform a test output in excess of 1,200 samples for nine analytes simultaneously per hour. Several independent studies already showed the validity of different Randox Evidence Multiplex assays [23-26]. For most of the markers it would be hard to find single ELISAs on the market using exactly the same antibodies. Thus, in order to test the overall validity of the multiplex chip assay, CEA, CRP, IL-8 and VEGF results for the CRCS multiplex array biochip were compared with commercially established Randox assays for individual markers using a minimum of 30 human serum samples. A good correlation was observed for all four markers tested, with r values > 0.95 (unpublished data). Therefore, this approach should become of increasing interest when considering limited sample volumes, costs, high-throughput, and reproducibility.

### Diagnostic performance of single markers and marker combinations

The effectiveness of any screening program depends not only on economic and operational viability of the screening test but also on excellent diagnostic performance. It should be noticed, that the prevalence of colon cancer and adenomas in our study group is higher than in the general population. However, our results indicate that multiplex biochip based serum protein profiling validated certain biomarkers to discern sera from patients with and without colon cancer. The analyses revealed combinations of CEA + IL-8 and CEA + CRP to show the best screening performance for colon cancer reaching a sensitivity of 47% and 39% in the validation set, respectively. These combinations also proved to be useful when applied to early carcinoma detection with 33% and 28% sensitivity, respectively, using the combined training and validation set. Applying the test algorithm to an additional independent sample set of sera from patients with colorectal adenomas, we could show a sensitivity of 18% and 15% sensitivity, respectively (both at 86% specificity). It would be highly desirable to directly compare the performance of individual markers both used here in a multiplex fashion and as single marker assay reported in the literature. Unfortunately, this comparison can be severely biased, reaching from different antibodies applied up to different sample processing and storage conditions. We therefore focused our analyses primarily on our multiplex results and those markers with the highest performance either alone or in combination.

CEA is a well-known serum marker linked to CRC [27]. It is the most commonly used and studied protein marker for diagnosis, prognosis, monitoring and recurrence after treatment of colorectal carcinomas [28]. CEA and other CAs tend to be elevated serum marker proteins also for other epithelial malignancies like pancreatic carcinomas [29]. Overall sensitivities for detecting CRC range from 43% to 69% as reviewed by Hundt *et al.*[22]. However, we now report for the first time the evaluation of CEA in combination with IL-8 while we could show a good diagnostic performance of IL-8 alone for detecting colon carcinomas previously [13]. In line, Fernandes *et al.* found that cytokeratins demonstrate a greater sensitivity than CEA in the diagnosis of colorectal carcinoma [30]. Lundberg *et al.* screened 148 patients regarding the expression of 74 putative biomarkers in plasma and reported among other markers CEA and IL-8 to be significantly elevated in CRC compared to healthy controls. They could hardly detect IL-8 by ELISA but by a proximity ligation-based multiplex assay [31]. A combination of CEA and CRP is rarely described: Stamatiadis *et al.* analyzed a combination of CEA and CRP for preoperative staging of colorectal cancer, however, not for early diagnosis or screening [32]. It has to be mentioned though that not a multiplex array but a retrospective statistical combination of separate assessments of both markers was used.

### Currently established screening test

Mortality from CRC can be reduced by early detection of cancer and removal of adenomas [33,34]. Based on this evidence, a number of countries have already introduced screening programs for CRC. Besides colonoscopy, the most common, non-invasive screening tool for colorectal cancer is fecal occult blood testing (FOBT). Several studies have shown that annual or biennial screening in asymptomatic people over the age of 50 years using FOBTs can reduce CRC mortality by 15–33% [35-37]. Diagnostic performance of FOBTs has greatly improved throughout the last 50 years. In 1986, Bang *et al.* reported a sensitivity of 25% at 98% specificity for detecting CRC [38]. In 1997, Ransohoff *et al.* reported 30 to 50% sensitivity at 84 to 96% specificity for FOBTs [39]. iFOBT displayed higher sensitivity of 61–91% for CRC and showed a clinically superior accuracy [40] at specificity varying from 91% to 98% [41].

#### Adenoma detection

Besides carcinomas, detection of early neoplasm like adenomas is favourable. For iFOBTs, sensitivities for detecting adenomas range from 4% to 63% for all adenomas, and 28% to 67% for adenomas of 1 cm or larger, both at a specificity of 89% [42]. Among the 34 adenoma patients included in our study were 12 early stage and 22 advanced adenomas [43]. The multiplex biochip showed a sensitivity of 18% for CEA + IL-8 and 15% for CEA + CRP, both at 86% specificity. While the detection of adenomas by serum protein markers might still be unexpected due to the relatively small lesion size and pre-malignant characteristics as compared to invasive carcinomas, our results fall into sensitivity ranges reported for various other approaches. In addition, the sensitivity of our biochip array can likely be improved by the addition of further promising markers.

#### Compliance of current screening methods

The effectiveness of any screening program depends not only on the diagnostic and economic performance of the screening test but also to a large extent on the compliance and general acceptance of the test by the public. Indeed, the compliance for colonoscopy is quite low compared to FOBTs [44]. Colonoscopy is thought to be time consuming, disturbing, painful and involving risk [45]. In contrast, 88.8% of patients reported that they would perform the fecal occult blood test (FOBT) for CRC screening if so requested by doctors [45]. If the FOBT was positive and a colonoscopy was offered, 84.9% of participants indicated that they would undergo the procedure [45]. Against this background it can easily be envisioned that a blood test would even reach comparable if not even a higher compliance compared to FOBT testing. While new screening technologies might enable detecting changes in bloods’ cfDNA and miRNA composition, proteins are more likely to present the actual disease phenotype. However, it would be desirable for future studies to compare the most potent screening assays including known markers, e.g. CEA and CA19-9.

## Conclusions

Our novel multiplex biochip revealed combinations of CEA + IL-8 and CEA + CRP to show the best screening performance for colon cancer with 47% sensitivity, for early carcinomas with 33% sensitivity, and adenomas with 18% sensitivity at an overall specificity of 86%. This performance could be improved by the addition of further promising markers. Since neither compliance nor diagnostic performance of FOBTs and serum markers alone seems satisfying for early colon cancer detection today, a combination of both methods may improve the performance of colon cancer screening.

## Competing interests

The authors declare that they have no competing interests.

## Authors’ contributions

SB and KKG were responsible for study group collection, performed the biochip analyses and drafted the manuscript. MK, AC, VT, DM and SPF were responsible for chip development and validation. UH was responsible for the statistical data analysis. TG and TL assisted in drafting the manuscript. NP participated in the project design. UJR, JB, KF and HPB participated in clinical sample collection. HB, FE, JKH conceived and coordinated the study and drafted the manuscript. All authors read and approved the final manuscript.

## Grant support

The consortium “Colon Cancer Screening Chip” was generously supported by the German Federal Ministry of Education and Research (BMBF) within the Molecular Diagnostics funding scheme (grants 01ES0720, 01ES0721, 01ES0722, and 01ES0723). The study was performed based on the serum collection of the biomaterial bank ColonBiomics being part of the Surgical Center for Translational Oncology – Lübeck (SCTO-L) and the “North German Tumorbank of Colorectal Cancer” network, the latter being generously supported by the German Cancer Aid Foundation (Dt. Krebshilfe e. V., grant #108446).

## Pre-publication history

The pre-publication history for this paper can be accessed here:

http://www.biomedcentral.com/1471-2407/12/393/prepub

## Supplementary Material

Additional file 1 **Table S1.** Summary of clinical data of the pilot study group. a) Summary of clinical data of the pilot study group consisting of 400 serum samples from DKFZ: colon cancer patients, pre-malignant adenoma patients and healthy control patients. *All healthy patients received a full colonoscopy. b) Summary of clinical data of colon cancer patients. Click here for file

Additional file 2 **Table S2.** Test performance of the multiplex-protein array. Assay ranges, intra- and inter-assay precision, accuracy and sensitivity of each analyte used on the CRCSI and CRCSII chip. *This is the full measuring range after dilution. Click here for file

Additional file 3 **Figure S1.** A) 6 Carrier Holder for simultaneously processing of 54 samples B) One Carrier with nine Biochip-Arrays, C) Randox Evidence Investigator and D) Screenshot of Analysis Software. Click here for file

Additional file 4 Instructions for use (IFU) for CRCSI and II. Click here for file

Additional file 5 **Table S3.** Summary of diagnostic performances of biomarkers. Summary of diagnostic performances of biomarkers in single or combined fashion for detection of colon carcinomas. nd: not defined. Click here for file

Additional file 6 **Table S4.** Spearman´s Correlation coefficient above 0.4. (up to 0.5, all with p < 0.001). Click here for file
