# Supplementary material for: A novel multiplex-protein array for serum diagnostics of colon cancer: a case–control study
Source: BMC Cancer. 2012 Sep 7;12:393. doi: 10.1186/1471-2407-12-393 (PMC3502594; doi:10.1186/1471-2407-12-393)
Supplement: Additional file 1 — Table S1. Summary of clinical data of the pilot study group. a) Summary of clinical data of the pilot study group consisting of 400 serum samples from DKFZ: colon cancer patients, pre-malignant adenoma patients and healthy control patients. *All healthy patients received a full colonoscopy. b) Summary of clinical data of colon cancer patients. [file 1471-2407-12-393-S1.docx]

**Additional file T1**

**a)** Summary of clinical data of the pilot study group consisting of 400 serum samples from DKFZ: colon cancer patients, pre-malignant adenoma patients and healthy control patients. **All healthy patients received a full colonoscopy*.

| **Parameter** | **Value** | **Colorectal cancer patients** | **Healthy**  **control patients** | **Early**  **adenoma patients** | **Advanced**  **adenoma patients** |
| --- | --- | --- | --- | --- | --- |
|  |  | **(n=100)** | **(n=100)*** | **(n=100)** | **(n=100)** |
| **Sex** | **Female** | 46% | 57% | 44% | 41% |
|  | **Male** | 54% | 43% | 66% | 59% |
| **Age (years)** | **Mean** | 68.3 | 63.8 | 65.1 | 63.9 |

**b)** Summary of clinical data of colon cancer patients

| **Parameter** | **Value** | **Colon cancer patients** |
| --- | --- | --- |
| **UICC stage** | 1 + 2 | 58% |
|  | 3 + 4 | 42% |
