# Supplementary material for: A novel multiplex-protein array for serum diagnostics of colon cancer: a case–control study
Source: BMC Cancer. 2012 Sep 7;12:393. doi: 10.1186/1471-2407-12-393 (PMC3502594; doi:10.1186/1471-2407-12-393)
Supplement: Additional file 2 — Table S2. Test performance of the multiplex-protein array. Assay ranges, intra- and inter-assay precision, accuracy and sensitivity of each analyte used on the CRCSI and CRCSII chip. *This is the full measuring range after dilution. [file 1471-2407-12-393-S2.docx]

**Additional file T3** Assay ranges, intra- and inter-assay precision, accuracy and sensitivity of each analyte used on the CRCSI and CRCSII chip. *This is the full measuring range after dilution

|  | **CRCSI** | | | **CRCSII** | | | | | |
| --- | --- | --- | --- | --- | --- | --- | --- | --- | --- |
|  | **CD26** ng/mL | **C3adesArg** ng/mL | **CRP**  ng/mL | **CEA**  ng/mL | **IL-8**  pg/ml | **VEGF** pg/mL | **S100A11**  ng/mL | **M-CSF** pg/mL | **NNMT** ng/mL |
| **Full Assay Range** | 0 – 4,000* | 0 – 120000 * | 0 – 60000* | 0 – 300 | 0 – 3,000 | 0 – 2,000 | 0 – 250 | 0 – 500 | 0 – 70 |
| **Precision – Intraassay** | ≤10% | ≤12% | ≤8% | ≤8% | ≤10% | ≤8% | ≤9% | ≤6% | ≤10% |
| **Precision –Interassay** | ≤9% | ≤14% | ≤8% | ≤12% | ≤8% | ≤14% | ≤14% | ≤7% | ≤12% |
| **Accuracy** | ≤95 - 103% | ≤79 - 84% | ≤94 - 103% | ≤91 -102% | ≤92 - 98% | ≤103 - 111% | ≤90 - 99% | ≤93 -100% | ≤79 - 90% |
| **Sensitivity** | 26 | 580 | 998 | 0.39 | 6.95 | 4.48 | 2.91 | 2.61 | 0.77 |
