# Supplementary figures and images for: A novel multiplex-protein array for serum diagnostics of colon cancer: a case–control study
Source: BMC Cancer. 2012 Sep 7;12:393. doi: 10.1186/1471-2407-12-393 (PMC3502594; doi:10.1186/1471-2407-12-393)

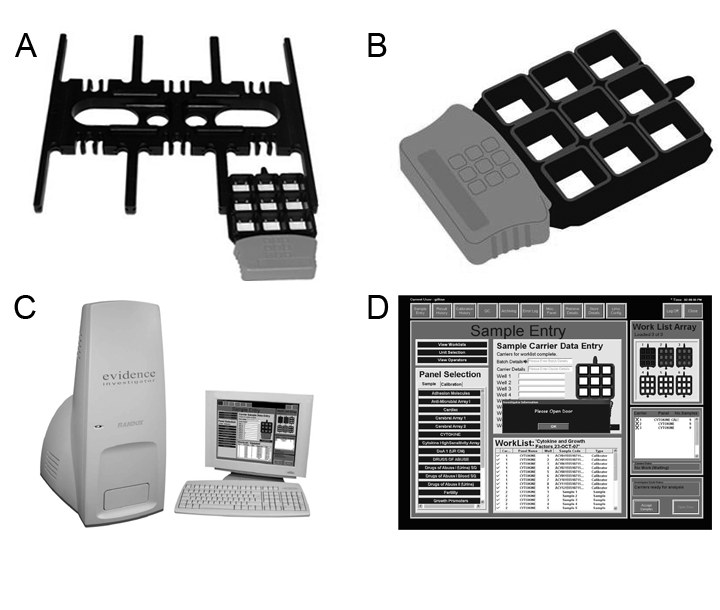

Supplement: Additional file 3 — Figure S1. A) 6 Carrier Holder for simultaneously processing of 54 samples B) One Carrier with nine Biochip-Arrays, C) Randox Evidence Investigator and D) Screenshot of Analysis Software. [file 1471-2407-12-393-S3.tiff]
