# Supplementary material for: A novel multiplex-protein array for serum diagnostics of colon cancer: a case–control study
Source: BMC Cancer. 2012 Sep 7;12:393. doi: 10.1186/1471-2407-12-393 (PMC3502594; doi:10.1186/1471-2407-12-393)
Supplement: Additional file 4 — Instructions for use (IFU) for CRCSI and II. [file 1471-2407-12-393-S4.docx]

**Instructions for use (IFU) for CRCSI and II**

1. Pipette 200 μl of assay diluent (for CRCSI) or 210 µL assay diluent (for CRCSII) per well.

2. Pipette 100 μl of calibrator/ control/ 1:200 diluted serum sample (10µL sample in 1990µL sample diluents, for CRCSI) or 90 µL undiluted serum (for CRCSII) per well.

3. Gently tap all edges of the handling tray to mix reagents.

4. Secure the handling tray to the base plate of the thermoshaker.

Incubate for 60 min at 37°C and 370 rpm.

5. Following 37°C incubation, discard reagents to waste using a sharp, flicking action of the handling tray. Immediately carry out 2 quick wash cycles. Using wash bottle with diluted wash buffer (refer to kit insert for dilution), add approx 350 μl wash buffer to each well, gently tap all edges of the handling tray to release any reagents trapped below the biochip, and flick to waste with a sharp action. Take care not to overfill wells during wash in order to reduce potential for well-to-well contamination. Carry out a further 6 wash cycles; for each cycle gently tap all edges of the handling tray for approximately 10 to 15 sec then leave the biochips to soak in wash buffer for 2 min.

6. After the final wash, tap the carrier gently onto lint free tissue to remove any residual wash buffer.

7. Immediately pipette 300 μl conjugate into each well and gently tap the handling tray.

8. Secure the handling tray to the base plate of the thermoshaker. Incubate for 60 min at 37°C and

370 rpm.

9. Following incubation, remove the handling tray containing carriers from the thermoshaker. Discard reagents to waste using a sharp, flicking action of the handling tray.

10. Immediately carry out 2 quick wash cycles. Using wash bottle with diluted wash buffer, add approx. 350 μl wash buffer to each well, gently tap all edges of the handling tray to release any reagents trapped below the biochip, and flick to waste with a sharp action. Take care not to overfill wells during wash in order to reduce potential for well to-well contamination. Carry out a further 6 wash cycles; for each cycle gently tap all edges of the handling tray for approximately 10 to 15 sec then leave the biochips to soak in wash buffer for 2 min.

11. After final wash, fill wells with wash buffer and leave to soak until directly prior to imaging. No carrier should be left to soak for longer than 30 min. Process carriers individually. Those awaiting imaging should be protected from light.

12. Remove the first carrier to be imaged from the handling tray. Directly before addition of signal, remove wash buffer using a sharp, flicking action and tap the carrier onto lint free tissue to remove any residual wash buffer.

13. Add 250 μl of working signal reagent to each well and cover to protect from light.

14. 2 min (+/-10sec) after addition of signal to the last well place the carrier inside the evidence Investigator, the use of a timer is recommended.
