# Supplementary material for: A novel multiplex-protein array for serum diagnostics of colon cancer: a case–control study
Source: BMC Cancer. 2012 Sep 7;12:393. doi: 10.1186/1471-2407-12-393 (PMC3502594; doi:10.1186/1471-2407-12-393)
Supplement: Additional file 5 — Table S3. Summary of diagnostic performances of biomarkers. Summary of diagnostic performances of biomarkers in single or combined fashion for detection of colon carcinomas. nd: not defined. [file 1471-2407-12-393-S5.docx]

**Additional file T4** Summary of diagnostic performances of biomarkers in single or combined fashion for detection of colon carcinomas.

nd: not defined.

|  | | **Single marker** | | | **Marker combinations** | | |
| --- | --- | --- | --- | --- | --- | --- | --- |
|  |  | **CRP**  ng/mL | **CEA**  ng/mL | **IL-8**  pg/ml | **CEA + IL-8** | **CEA + CRP** | **CEA + IL-8 + CRP** |
| **Trainingset** | Sensitivity (%) | 17 | 26 | 22 | 37 | 35 | 41 |
|  | Specificity (%) | 90 | 90 | 90 | 83 | 81 | 73 |
| **Validationset** | Sensitivity (%) | nd | 37 | nd | 47 | 39 | nd |
|  | Specificity (%) | nd | 86 | nd | 86 | 86 | nd |
