# Supplementary material for: A novel multiplex-protein array for serum diagnostics of colon cancer: a case–control study
Source: BMC Cancer. 2012 Sep 7;12:393. doi: 10.1186/1471-2407-12-393 (PMC3502594; doi:10.1186/1471-2407-12-393)
Supplement: Additional file 6 — Table S4. Spearman´s Correlation coefficient above 0.4. (up to 0.5, all with p < 0.001). [file 1471-2407-12-393-S6.docx]

**Additional file T2 -** Spearman´s Correlation coefficient above 0.4 (up to 0.5, all with p<0.001)

|  | **CEA** | **IL-8** | **VEGF** | **M-CSF** | **S100A11** | **NNMT** | **C3adA** | **CRP** |
| --- | --- | --- | --- | --- | --- | --- | --- | --- |
| **CEA** | n.a. |  |  |  |  |  |  |  |
| **IL-8** |  | n.a. |  |  | 0.466 |  |  | 0.492 |
| **VEGF** |  |  | n.a. |  |  |  |  |  |
| **M-CSF** |  |  |  | n.a. |  |  |  | 0.406 |
| **S100A11** |  | 0.466 |  |  | n.a. |  |  |  |
| **NNMT** |  |  |  |  |  | n.a. |  |  |
| **C3adA** |  |  |  |  |  |  | n.a. |  |
| **CRP** |  | 0.492 |  | 0.406 |  |  |  | n.a. |
